# Study on chromatin regulation patterns of expression vectors in the PhiC31 integration site

**DOI:** 10.1080/15592294.2024.2337085

**Published:** 2024-04-09

**Authors:** Xueli Liu, Qina Chen, Xudong Yin, Xiao Wang, Jinshan Ran, Wei Yu, Bin Wang

**Affiliations:** a Key Technology Engineering Center for New Veterinary Vaccine and Industry of Yunnan Provincial Education Department, Kunming University, Kunming, Yunnan, China; b Pharmaceutical Department, Second Affiliated Hospital of Xinxiang Medical University, Xinxiang, Henan, China; c Department of Pharmacy, 920th Hospital of Joint Logistics Support Force, PLA, Kunming, China

**Keywords:** PhiC31 integration site, nucleosome-free region, activating RNA, ubiquitous chromatin-opening element, pseudo attp site

## Abstract

The PhiC31 integration system allows for targeted and efficient transgene integration and expression by recognizing pseudo attP sites in mammalian cells and integrating the exogenous genes into the open chromatin regions of active chromatin. In order to investigate the regulatory patterns of efficient gene expression in the open chromatin region of PhiC31 integration, this study utilized Ubiquitous Chromatin Opening Element (UCOE) and activating RNA (saRNA) to modulate the chromatin structure in the promoter region of the PhiC31 integration vector. The study analysed the effects of DNA methylation and nucleosome occupancy changes in the integrated promoter on gene expression levels. The results showed that for the OCT4 promoter with moderate CG density, DNA methylation had a smaller impact on expression compared to changes in nucleosome positioning near the transcription start site, which was crucial for enhancing downstream gene expression. On the other hand, for the SOX2 promoter with high CG density, increased methylation in the CpG island upstream of the transcription start site played a key role in affecting high expression, but the positioning and clustering of nucleosomes also had an important influence. In conclusion, analysing the DNA methylation patterns, nucleosome positioning, and quantity distribution of different promoters can determine whether the PhiC31 integration site possesses the potential to further enhance expression or overcome transgene silencing effects by utilizing chromatin regulatory elements.

## GRAPHICAL ABSTRACT

**Figure:**
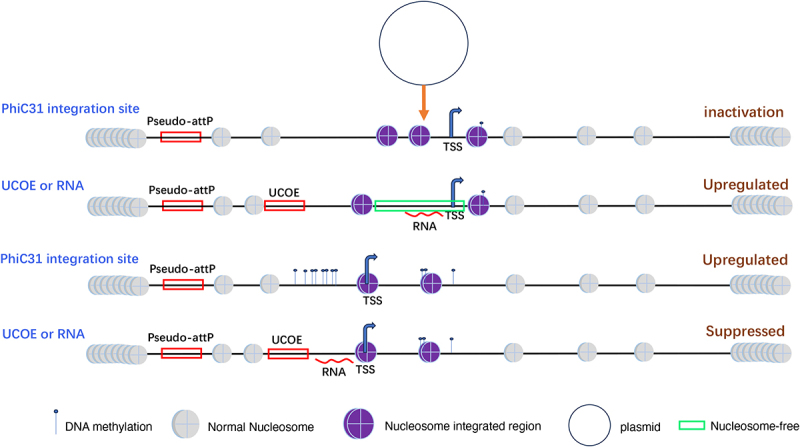

## Introduction

In the pharmaceutical industry, the efficient expression of exogenous genes in mammalian cells is an essential pre-requisite for gene engineering [1]. However, current mammalian cell expression systems suffer from certain limitations. These are low protein expression, unstable cell lines, and high costs [2]. These challenges can be addressed by using PhiC31 site-specific integrase, which recognizes pseudo-attP sites in mammalian cells and facilitates site-specific recombination, thereby resulting in stable, long-term, and high-level transgene expression [3,4]. Compared with random integration, PhiC31 phage-mediated integration achieves a five- to ten-fold higher efficiency in HEK293 and 3T3 cells [5]. This system can, for example, be used to enhance the expression of monoclonal antibodies in CHO cells, leading to higher titres and enhanced protein stability, and thereby highlighting its utility as a powerful tool for antibody production in recombinant CHO cell lines [6,7]. Most PhiC31 sites are located in active transcription regions, mainly distributed in intergenic regions and introns of the genome, with only a few being found in exons [8,9]. PhiC31 integrase-mediated gene therapy has been established to have no adverse effects in animals [10–13], thus indicating its potential application as an effective and safe integration system for mammalian cell expression vectors.

PhiC31 phage integrase efficiently integrates exogenous plasmids into readily expressed open chromatin regions of mammalian chromosomes [14]. By mediating recombination between the PhiC31 attB site and pseudo-attP sites in the mammalian genome, it achieves autonomous, efficient, unidirectional, and site-specific integration into the host genome [15,16]. Despite this efficacy, however, not all PhiC31 integration sites provide optimal structures for the high expression of exogenous genes. By combining CRISPR/Cas9 technology [17,18] with nuclear localization signals [19], a specific integration site can be selected from multiple pseudo-attP sites to achieve efficient transgenic expression. So, when using pseudo-attP-mediated integration, there are notable variations in the efficiency with which exogenous genes integrated into different open chromatin regions are expressed. Furthermore, the integrated open chromatin region remains susceptible to transgene silencing effects [20,21]. To overcome the adverse effects of transgene silencing, it is typically necessary to incorporate regulatory elements into the expression vector that can prevent both transgene silencing and position effects, this ensures a more efficient and stable integrated expression [22–24]. Additionally, modulation of the expression vector’s promoter through the use of chromatin regulatory elements or activating RNA can further enhance expression efficiency and impede transgene silencing by modifying the chromatin structure [25,26]. However, it remains to be clarified whether the open chromatin region integrated by PhiC31 can be regulated by chromatin regulatory elements and RNA to further improve expression and overcome transgene silencing effects.

In this study, we utilize Ubiquitous Chromatin Opening Element (UCOE) [27–28], which can prevent DNA methylation and alter chromatin structure, along with small activating RNA (saRNA) [25–26] to regulate the open chromatin region of the promoter in the PhiC31 integration vector. We analyse the relationship between DNA methylation, nucleosome occupancy changes in the open chromatin region integrated by PhiC31, and expression levels. By investigating the regulation of efficient expression and overcoming transgene silencing in the open chromatin region integrated by PhiC31, this research aims to provide new insights for the industrial application of the PhiC31 integration system in mammalian cells.

## Materials and methods

### Vector construction

In this study, we have previously constructed eight vectors: pOCT4, pSOX2, pOCT4-UCOE, pSOX2-UCOE, pOCT4-O38, pSOX2-S278, pOCT4-UCOE-O38, and pSOX2-UCOE-S278. The functionality of each vector has been validated. Among them, the saRNA-regulated pOCT4-O38 vector contains a targeting saRNA expression cassette downstream of the OCT4 gene promoter (ENSG00000204531, see attachment for the selected sequence) in the eukaryotic expression framework of the pOCT4 vector. Similarly, the pSOX2-S278 vector contains a targeting saRNA expression cassette downstream of the SOX2 gene promoter (ENSG00000181449, see attachment for the selected sequence) in the eukaryotic expression framework of the pSOX2 vector [26]. The sequences for O38 and S278 are CGCCTTTAATCATGACACT and GCACCTGTAAGGTAAGAGA, respectively.

The pOCT4-UCOE-O38 and pSOX2-UCOE-S278 vectors were generated by adding a 1.5kb UCOE fragment [27] (see attachment) to the pOCT4-O38 and pSOX2-S278 vectors, respectively, for regulation. These vectors enable co-regulation of the target promoters OCT4 and SOX2 by UCOE and saRNA. To facilitate integration into the PhiC31 integration region, the attB plasmid element (sequence: GTGCCAGGGCGTGCCCTTGGGCTCCCCGGGCGCG) of the PhiC31 integration system were inserted upstream of the respective promoters and UCOE regulatory elements in all eight vectors. The vectors were named as follows: pOCT4-attB (abbreviated as pOB), pSOX2-attB (abbreviated as pSB), pOCT4-UCOE-attB (abbreviated as pOUB), pSOX2-UCOE-attB (abbreviated as pSUB), pOCT4-O38-attB (abbreviated as pOOB), pSOX2-S278-attB (abbreviated as pSSB), pOCT4-UCOE-O38-attB (abbreviated as pOUOB), and pSOX2-UCOE-S278-attB (abbreviated as pSUSB). All these vectors were sequenced and validated by Beijing Qingke Biotechnology Co., Ltd.

### Cell culture and monoclonal cell line screening

CHO-K1 cells were cultured in RPMI 1640 medium supplemented with 10% foetal bovine serum. On reaching 90% confluence, the cells were dissociated using 0.25% trypsin and seeded in 24-well plates at a density of 2 × 10^5^ cells per well. Having reached 80% confluence, cell transfection was performed using EntransterTM-D4000 transfection reagent (4000219112320201001; Engreen Biosystem Ltd). Plasmid vectors without the PhiC31 integration system attB fragment were directly transfected into CHO-K1 cells, whereas vectors carrying attB were co-transfected with a pCMV-Int plasmid which containing the PhiC31 integrase gene (Hunan Fenghui Biotechnology Co., Ltd, Hunan, China). Following transfection, the cells were incubated at 37°C within a 5% CO_2_ incubator. Selection was performed by adding 750 µg/mL G418 to the culture medium and culturing continuously for 20 days to obtain stably growing transfected cells. Monoclonal cell line screening was subsequently performed using the soft agar method.

### Western blotting

Monoclonal cell lines were cultured in T25 culture flasks until reaching 90% confluence. At this point, cell proteins were extracted using NP-40 lysis buffer (P0013F; Beyotime) and concentrations were determined using a BCA protein assay kit (P0010; Beyotime). Aliquots (20 µg per lane) of the extracted proteins were run on 12% SDS-PAGE gels, and the separated proteins were transferred to membranes using the wet transfer method. Following transfer, the membranes were incubated at room temperature with blocking solution containing 5% BSA (4240GR100; BioFROXX) for 1 h. Subsequently, the membranes were incubated overnight at 4°C with primary antibodies, namely, EGFP antibody (66002; Proteintech) and β-actin antibody (66009–1-1 g; Proteintech). The following day, the membranes were incubated with secondary antibody (152551; Proteintech) at room temperature for 1 h. Protein bands were visualized using an ECL detection system (A38555;ThermoFisher scientific) and exposed.

### Quantitative real-time PCR

Monoclonal cell lines were cultured in T25 culture flasks until reaching confluence, at which point, 5 × 10^6^ cells were harvested. Total RNA was extracted using a total RNA extraction kit (LS1040; Promega). The extracted RNA was reverse transcribed to cDNA using a reverse transcription kit (G492; abm). CHO wild-type cells were used as the control group, GAPDH (primer sequence: AAGGTCGGCGTGAACGGATTT and CCCATTTGATGTTGGCGGGAT) was used as the reference gene, and EGFP (primers: CCACAAGTTCAGCGTGTCCGGCGAG and ACGTAGCCTTCGGGCATGGCGGAC) served as the target gene. Real-time quantitative PCR (qRT-PCR) was performed in an ABI 7500 fast thermal cycler using EvaGreen 2× qPCR MasterMix (G891; abm). The amplification programme incorporated an initial denaturation step at 95°C for 5 min, followed by 40 cycles of denaturation at 95°C for 15 s and annealing/extension at 60°C for 1 min. Three replicate analyses were performed for each sample. The relative levels of EGFP expression among samples were determined using the 2^-(∆∆CT) method. Perform statistical analysis using GraphPad Prism 8 software. Use the T-test method to compare two samples, and use the ANOVA method to analyse the differences in EGFP expression levels among multiple samples.

### Analysis of nucleosome deletion

Following the procedure described by Wang et al. [26], we performed nucleosome deletion analysis on individual monoclonal cell lines, for which we used approximately 2 × 10^6^ cells. Chromatin was cleaved at each nucleosome using 0.25 µL of Micrococcal Nuclease (ChIP Grade) (10 U/µL). A fraction of the resulting chromatin was reserved as the input control. Immunoprecipitation (IP) of nucleosome-associated DNA was carried out using a Histone H2A monoclonal antibody (ChIP Grade) (Cell Signaling Technology, Inc, D6O3A), yielding the experimental IP sample.

For qRT-PCR analysis, we performed a comparison between the IP sample and the input control, with *GAPDH* serving as the reference gene. The OCT4 promoter region was divided into 12 segments for continuous analysis, whereas the SOX2 gene promoter region was partitioned into 10 segments. A total of 12 primer pairs were designed, targeting 100-bp intervals within the OCT4 promoter region, and 10 primer pairs were designed for screening the SOX2 promoter region (see Supplementary Table S1). Regions exhibiting an amplification ratio [2^(-∆∆CT) value] below 0 in the IP sample compared to the input sample were identified as nucleosome-depleted regions. Conversely, regions showing higher or no differential expression in the IP sample relative to the input sample (2^(-∆∆CT) value) were classified as nucleosome-covered regions. A continuous distribution curve was plotted to visualize the relative expression levels of each primer segment on the respective promoters. Peaks on the curve represent primer segment target locations with an IP-to-input ratio greater than 0, indicating nucleosome occupation. Valleys on the curve indicate targeted primer segment locations showing an IP-to-input ratio of less than 0, indicating the presence of nucleosome-depleted regions.

### Nucleosome occupancy and methylation sequencing (NOME-seq)

Using the method described by Lay et al. [28], we prepared 2 million test cells, which were lysed using a 0.5% NP-40 lysis buffer (P0013F; Beyotime) containing 1% PMSF at 4°C for 5 to 6 s. The lysates were washed twice with PBS (containing 1% PMSF) and cell nuclear precipitates were collected by centrifugation at 2500 × ***g*** and 4°C using a nuclear extraction kit. Sample of these preparations (containing 1 million cell nuclei) were mixed with 5 μL of 10× buffer, 0.75 μL of 32 mM SMA, 5 μL of GC methyltransferase, 45 μL of sucrose, and water up to a total volume of 150 μL. Having allowed the reaction to proceed for 10 min, a further 5 μL of GC methyltransferase and 0.75 μL of SAM were added and the reaction was continued for a further 10 min, after which, the reaction was terminated by heating at 65°C for 20 min. The suspensions were thereafter centrifuged at 10,000 × ***g***. Having discarded the supernatants, the resulting cell nuclei precipitates were extracted using a DNA extraction kit (batch number: 74702445; Beijing Biomed Co., Ltd.,). The extracted DNA was subjected to bisulphite conversion using an ZYMO EZ DNA Methylation-Gold Kit™ (D5001; Zymo, U.S.) to convert all unmethylated C to T. The converted DNA was purified using the DNA Wizard SV Gel and PCR Clean-Up System (batch number: 0000266856; Promega) and analysed for bisulphite sequencing (illumina Xten, Shenzhen Yi Gene Technology Co., Ltd).

### Analytic methods of NOME-seq

The deep sequence raw data was first filtered using adapter sequences ‘-a AGATCGGAAGAGC -m 35 -n 2’ (see Supplementary Table S2-S3). The resulting cleaned reads were then mapped back to the genome using BSMAP software version 2.90 [29]. The specific parameter settings used were ’-n 0 -v 0.08 -g 1.’ Methylation ratios were obtained from the BSMAP output (SAM) using a Python script called ‘methratio.py’ included in the BSMAP package. Only uniquely mapped reads were utilized for calculating methylation ratios. Furthermore, only cytosines in a CpG and GpC context were retained for further analysis if they had sufficient sequencing depth (greater than or equal to 5× coverage).

For determining CG methylation and GC methylation, a methylation level of ≥ 0.6 was considered. To identify samples treated with regulatory elements (such as pSS, pOO, etc.), pS and pO were used as negative controls. Using the analytical procedure described by Requena F et al. [30], the methylated regions were further divided into the following subcategories: regions without GC methylation between 150 and 200 bp were defined as one-nucleosome coverage region; regions without GC methylation between 80 and 120 bp were defined as transcription factor coverage regions; regions without GC methylation between 120 and 140 bp were defined as small nucleosome coverage regions; regions without GC methylation between 200 and 300 bp were defined as large nucleosome coverage regions; regions without GC methylation between 300 and 400 bp were defined as two-nucleosome clustering regions; and regions without GC methylation between 400 and 550 bp were defined as three-nucleosome clustering regions.

## Results

### The PhiC31 system enhances the stability of cell cloning and expression of integrated vectors

**Figure 1.**
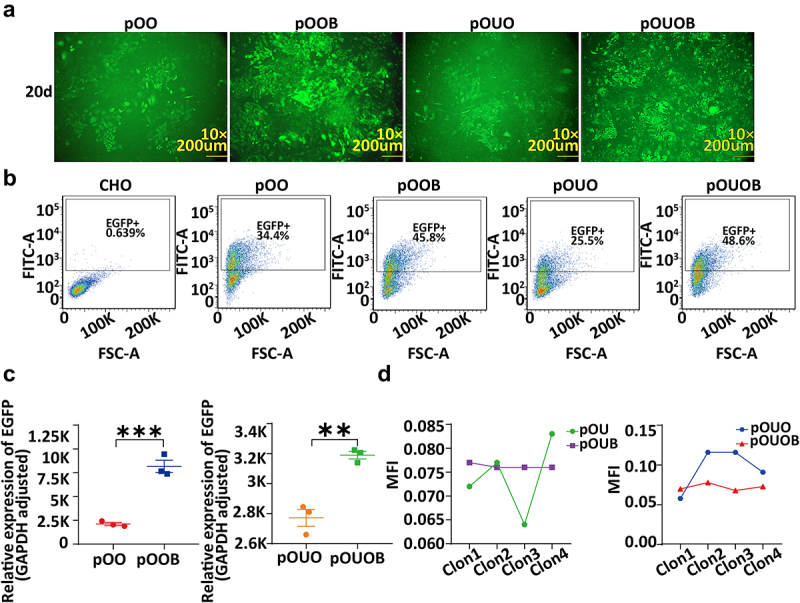
Expression and stability of the EGFP gene in cell clones integrated with PhiC31 and non-PhiC31 integration vectors, analysed using fluorescence intensity, flow cytometry, and qRT-PCR. (a) Fluorescence microscopy images of four cell clones. (b) Flow cytometry analysis results of the four vector cell clones. (c) qRT-PCR analysis of the EGFP gene expression levels for the four vectors. (d) Analysis of fluorescence intensity to assess stable EGFP expression for the four vectors.

The plasmids pOOB and pOUOB were introduced into CHO cells using the PhiC31 integration system, and these cells were compared with those transformed with the pOO and pOUO vectors lacking the attB site. On the 20^th^ day of stable post-transfection growth, we analysed expression of the reporter gene EGFP in the cell clones containing the integrated constructs using a combination of approaches, namely, average fluorescence intensity, flow cytometry, and qRT-PCR analyses (Figure 1(a–c)). The results indicated that compared with the non-PhiC31-integrated pOO vector cell clones, the cell clones carrying the PhiC31-integrated pOOB vector exhibited significantly higher levels of EGFP expression (*t*-test, *p* < 0.0005) (Figure 1(c)). Moreover, when the two cell clones were regulated using UCOE, those clones carrying the PhiC31-integrated pOUOB vector also showed significantly higher levels of expression than those with the non-directed integration of the pOUO vector (*t*-test, *p* < 0.005) (Figure 1(d)).

Using a flow cytometry cell sorter, cell clones transfected with the aforementioned vectors (with EGFP as the reporter gene) were analysed for those displaying higher average fluorescence intensity after 20 days (Figure 1(b)). The results revealed that among the stably transfected cell populations harbouring the pOO, pOOB, pOUO, and pOUOB vectors, the percentages of cells clustering in the high average fluorescence intensity group were 34.4%, 45.8%, 25.5%, and 48.6% respectively, indicating that pOOB > pOO and pOUOB > pOUO. These findings indicate that compared with random integration, PhiC31 site-specific integration contributed to a significant increase in the proportion of cells with stable transfection and high expression levels. Following stable transfection with the pOUO, pOUOB, pOU, and pOUB plasmids, four different monoclonal cell lines were randomly selected. Fluorescent inverted microscopy images were captured, and the average fluorescence intensities (MFIs) of each clone were analysed using ImageJ software (Figure 1(a–d)). The results revealed minimal differences in MFI values between the single-cell clones stably transfected with PhiC31-integrated pOUB and pOUOB vectors, whereas, we detected significant differences in the MFI values of the clones stably transfected with the pOUO and pOU vectors(*t*-test, *p* < 0.05), indicating that compared with non-integrated clones, gene expression levels among single-cell clones integrated with the PhiC31 system were more stable.

### High expression mediated by the PhiC31 system and its association with the promoters of integrated vectors

**Figure 2.**
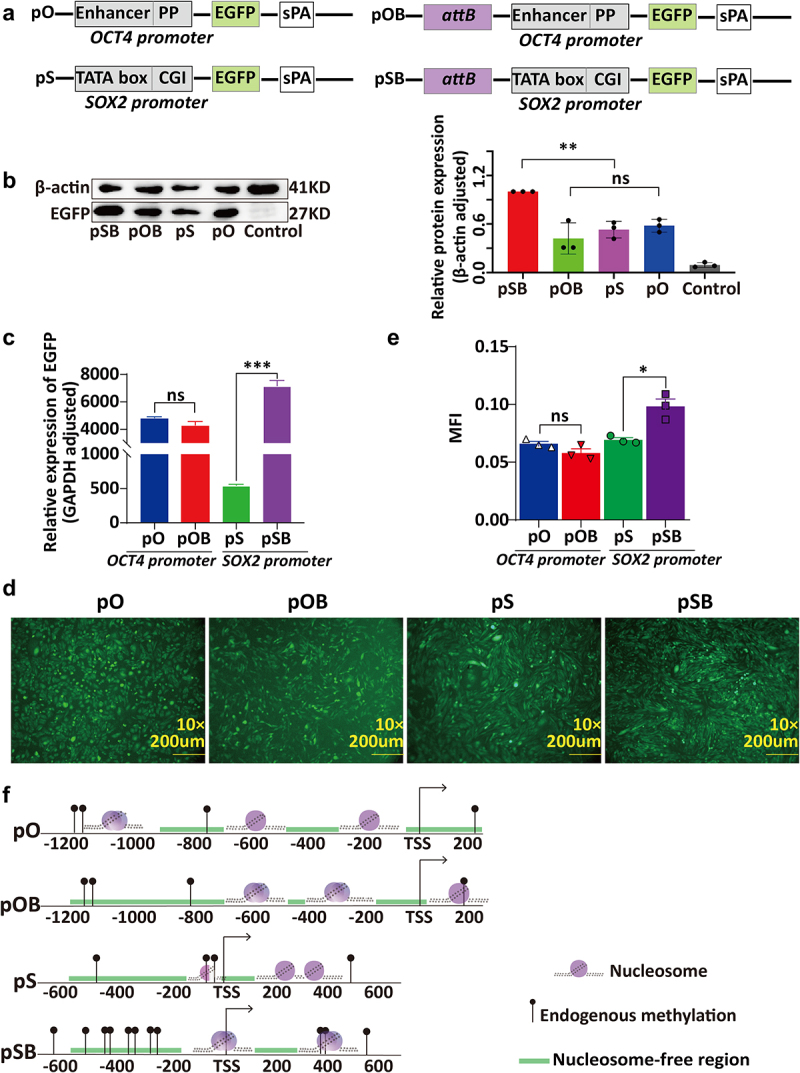
The relationship between chromatin structure changes in the promoter regions of PhiC31-integrated vectors and their expression levels. (a) Structure of the four vectors, pO, pOB, pS, and pSB. (b) Western blot analysis of EGFP expression in the cell clones containing the four vectors.(c) qRT-PCR analysis of EGFP expression in the cell clones containing the four vectors. (d) Fluorescence microscopy images of the cell clones containing the four vectors. (e) ImageJ analysis of fluorescence intensity in the cell clones containing the four vectors. (f) Schematic representation of chromatin structure in the promoter regions of the four vectors obtained based on NOMe-seq analysis.

In order to investigate the regulatory patterns of different promoters in the PhiC31 integration region, we selected the OCT4 gene promoter with a medium CG density and enhancer and the SOX2 gene promoter with a high CG density and two CpG islands as control experiments. In differentiated cells, both the OCT4 and SOX2 genes were almost non-expressed. Using these two promoters, we constructed pO (pOCT4), pSOX2 (pSOX2), and their attB-containing counterparts pOB and pSB (Figure 2(a)). pOB represented the open chromatin region integrated with the OCT4 promoter containing a lower CG density and enhancer region, while pSB represented the insertion of the CG-rich SOX2 promoter into the open chromatin region. These four vectors were individually transfected into CHO cells to generate monoclonal cell lines. The expression of EGFP carried by each vector was examined in these cells using western blot, qRT-PCR, and average fluorescence intensity analyses. The results revealed that although there were no significant differences in expression between cells transfected with the pO and pOB vectors (t-test, *p* > 0.05), the levels of EGFP expression in cells transfected with the pSB vector were significantly higher than those carrying the pS vector (t-test, *p* < 0.05) (Figure 2(b–e)).

To establish the factors contributing to the PhiC31 system-mediated increase in expression under the control of the SOX2 promoter and the unaltered expression from the OCT4 promoter, we performed NOMe-seq analysis to examine the changes in chromatin structure in the OCT4 and SOX2 promoter regions among the four vectors (Figure 2(f)). The results revealed that whereas the DNA methylation levels and positions in the OCT4 promoter integrated with the PhiC31 system (pOB) remained largely unchanged, significant changes were observed in the locations of nucleosome-free regions and nucleosomes. Specifically, we detected a large (500 bp) nucleosome-free region between −1100 bp and −600 bp in the PhiC31 integration region of the OCT4 promoter. Notably, nucleosomes located upstream (−200 bp) of the transcription start site (TSS) in the pO OCT4 promoter were found to have been shifted downstream to the + 200 bp position. Similarly, substantial changes in nucleosome organization were observed in the PhiC31-integrated SOX2 promoter (pSB), including complete DNA methylation within the region from 200 bp to 400 bp upstream of the TSS and a nucleosome-free region from 0 to 400 bp downstream of the TSS.

### Regulation of PhiC31 integration site vector expression by UCOEs

**Figure 3.**
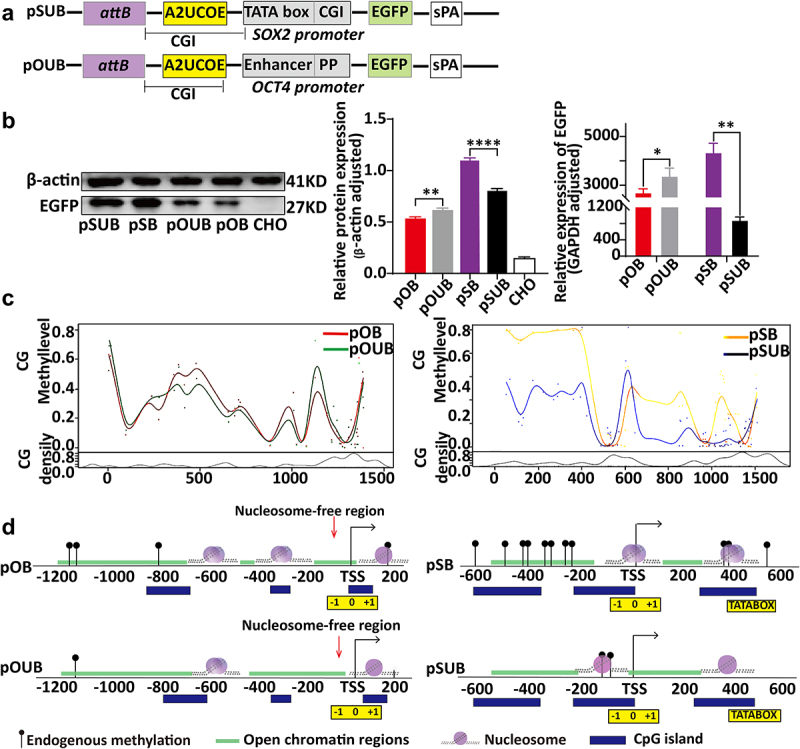
Relationship between ubiquitous chromatin-opening element (UCOE) regulation, chromatin structural changes, and expression levels of PhiC31 integration site sectors. (a) Structure of ubiquitous chromatin-opening element (UCOE)-regulated vectors. (b) Results of western blot and qRT-PCR analyses showing EGFP expression levels for the four vectors. (c) Changes in DNA methylation in the OCT4 and SOX2 promoter regions within the PhiC31 integration site following UCOE regulation. (d) Analysis of changes in chromatin structure in the PhiC31 integration site before and after UCOE regulation based om NOMe-seq analysis.

To investigate the effects of UCOE regulation on the expression of different promoter regions within the PhiC31 integration site, a 1.5-kb A2UCOE was inserted between the upstream region of the promoter and the downstream region of the attB site in the pOB and pSB vectors, resulting in the construction of vectors pOCT4-UCOE-attB and pSOX2-UCOE-attB (abbreviated as pOUB and pSUB, respectively) (Figure 3(a)).

The PhiC31 integration system was used to integrate pSUB and pOUB into CHO cells to obtain monoclonal cell lines.Analysis using western blotting, qRT-PCR, flow cytometry, and average fluorescence intensity showed that the expression level of the pOUB vector was significantly higher compared to pOB. On the other hand, the expression of pSUB was significantly lower than that of pSB (Figure 3(b)). Additionally, our NOMe-seq analysis indicated that the pOB and pSB vectors were subject to UCOE-mediated regulation of DNA methylation. The results indicated the pOUB vectors had two fewer methylated sites compared to the pOB vectors, whereas compared with pSB, methylation levels were significantly reduced in pSUB (Figure 3(c)).Compared to the non-PhiC31-integrated pOU vector, the pOUB vector exhibited a reduction of one nucleosome on the OCT4 promoter, accompanied by a significant increase in the width of the open chromatin region upstream of the transcription start site (TSS). The positioning of nucleosomes on the pSUB vector did not show significant changes. However, on the pSB vector, two large nucleosomes covering regions larger than 200bp (referred to as ‘large nucleosome regions’) were replaced by two normal nucleosome regions found in pSUB, with almost no change in the position of open chromatin. Notably, the methylated region in the open chromatin area upstream of the SOX2 promoter TSS completely disappeared in the pSUB vector (Figure 3(d)).

### Activation of RNA-mediated changes in chromatin structure in PhiC31 integration site promoters

**Figure 4.**
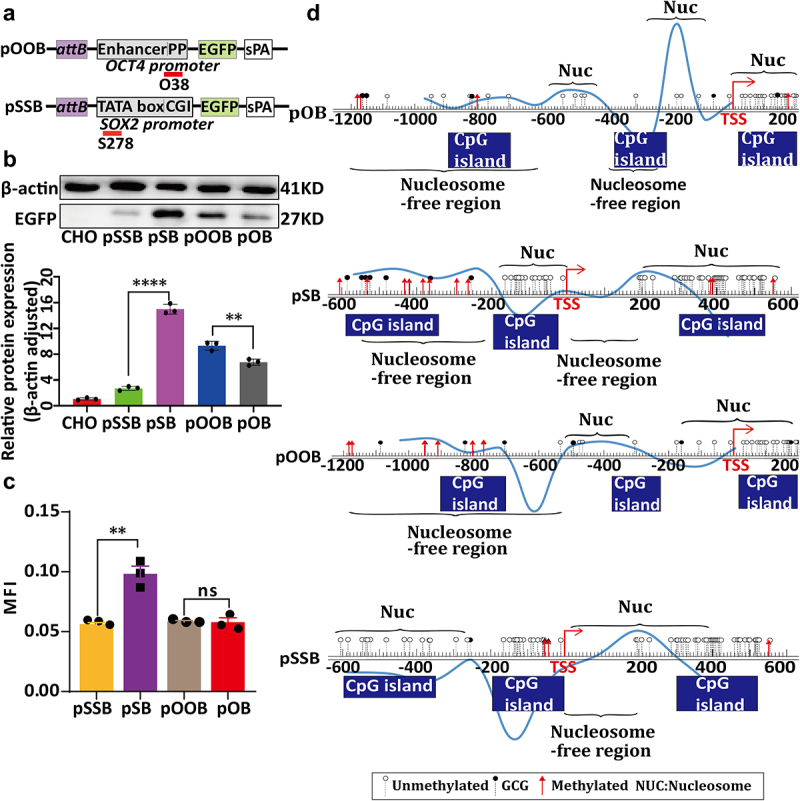
Analysis of relative expression levels and chromatin structure in small activating RNA (saRNA)-targeted PhiC31 integrated vectors containing OCT4 and SOX2 promoters. (a) A Schematic representation of saRNA targeting the promoters of PhiC31 integrated vectors. (b and c) Analysis of the effect of saRNA targeting on EGFP expression levels using western blotting.(d) Analysis of the average fluorescence intensity of EGFP in saRNA targeted PhiC31 integrated vectors. (e) Combined visualization of chromatin structure changes using NOMe-seq and nucleosome deletion analyses, represented by curve plots and site maps.

To further investigate changes in chromatin structure, we used saRNAs to target and activate the OCT4 and SOX2 promoters within the PhiC31 integration sites, and examined their effects on the expression of different promoters within the PhiC31 these sites. We accordingly detected changes in open chromatin and expression of the downstream EGFP gene. saRNA O38 and S278 shRNA expression cassettes were separately constructed downstream of the OCT4 and SOX2 promoters in pOB and pSB vectors, yielding the vectors pOCT4-O38-attB and pSOX2-S278-attB (abbreviated as pOOB and pSSB), respectively (Figure 4(a)).

The PhiC31 integrase-mediated pSSB and pOOB vectors were used to transfect CHO cells, and monoclonal cell lines were screened. Levels of the relative expression of the vector-encoded EGFP protein were analysed using western blotting (Figure 4(b,c)). In addition, we obtained inverted fluorescence microscopy images and average fluorescence intensity (MFI) values of the vectors using ImageJ software (Figure 4(d)). The results revealed that the relative levels of EGFP protein expression in cells harbouring the pSSB vector were significantly lower than those in cells transfected with the pSB vector, with the difference being highly significant (*p* < 0.0005). Contrastingly, whereas we detected no significant difference between the pOOB and pOB vectors with respect to MFI values (*p* > 0.05), western blot results indicated that the EGFP protein expression mediated by the pOOB vector was significantly higher than that mediated by the pOB vector.

The combination of NOME-seq and nucleosome deletion methods enables the derivation of nucleosome positions. The analytical results of nucleosome deletion are represented by a curve line. Peaks on the curve indicate the presence of nucleosomes, while valleys indicate regions where nucleosomes have been removed. By cross-referencing both methods, the locations of nucleosomes can be determined. The findings revealed that DNA methylation in the promoter region of the pOOB vector did not show significant changes when compared to the pOB vector. However, we detected the absence of a large nucleosome spanning the −700 bp to −500 bp region of the promoter, resulting in a nucleosome-free region of 200 bp width. Similarly, we detected the removal of a further large nucleosome from −400 bp to −170 bp region, leading to the formation of open chromatin adjacent to the TSS. In contrast, when comparing the pSSB and pSB vectors, we detected a significant reduction in DNA methylation in the SOX2 promoter region. In addition, the extent of open chromatin upstream of the TSS was found to have widened, whereas open chromatin downstream of the TSS had disappeared, resulting in the formation of two nucleosome-enriched ‘barrier’ points (Figure 4(e)).

### Simultaneous regulation of the PhiC31 integration site promoter by UCOEs and saRNA

**Figure 5.**
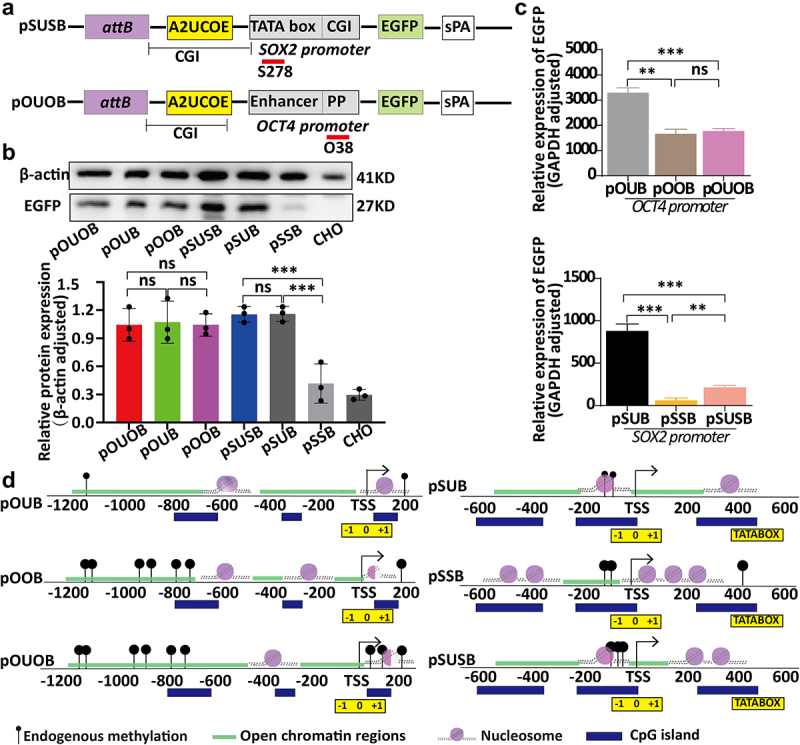
Changes in chromatin structure and effects on expression levels induced by simultaneous targeting of PhiC31 integration site promoters using ubiquitous chromatin-opening elements (UCOE) and small activating RNAs (saRnas). (a) Structural representation of vectors in which UCOE and saRNA co-regulate the same promoter. (b and c) Analysis of the effects of UCOE and saRNA co-regulation on EGFP expression levels using western blotting and qRT-PCR methods.(d) a schematic representation of chromatin structure changes in the vectors analysed based on NOMe-seq.

In order to investigate the simultaneous regulation of chromatin structure in the PhiC31 system integration site promoters by UCOEs and saRNA, we constructed expression vectors pOUOB and pSUSB incorporating both UCOEs and saRNA (Figure 5(a)).

The pOUOB and pSUSB vectors were co-transfected with the pCMV-Int vector into CHO cells, and monoclonal cell lines were selected. Expression of the EGFP gene encoded by the vectors pOUB, pOOB, pOUOB, pSUB, pSSB, and pSUSB was analysed using western blotting and qRT-PCR, the relative expression from qRT-PCR showed that the OCT4 promoter within the PhiC31 integration site was characterized by the highest expression when regulated by UCOE alone (pOUB vector), with significant differences observed compared with the levels of expression from pOOB regulated by saRNA alone (*p* < 0.005) and from pOUOB under saRNA – UCOE co-regulation (*p* < 0.0005). However, we detected no significant difference in expression between three vectors from western blotting. The SOX2 promoter within the PhiC31 integration site exhibited the highest expression when regulated by UCOE, with significant differences being observed compared with the expression from the pSSB and pSUSB vectors (*p* < 0.0005), Similarly, the result from western blotting and qRT-PCR between pSUB and pSUSB were different. Furthermore, in response to saRNA – UCOE co-regulation, we detected a significantly higher level of expression from the pSUSB vector than from the pSSB vector (*p* < 0.005) (Figure 5(b,c)).

Analysis of chromatin accessibility using two different methods in the aforementioned vectors incorporating an integrated PhiC31 system indicated that DNA methylation status in the OCT4 promoter of the pOUOB vector was highly consistent with that of the pOOB vector. Compared with pOUB, both pOUOB and pOOB showed a significant increase in DNA methylation in the upstream open chromatin region near the TSS. In terms of nucleosome changes, the nucleosome-free region near the TSS in the OCT4 promoter of the pOUB vector was significantly wider than that in the pOUOB and pOOB vectors. These findings tend to indicate that DNA methylation and the width of the nucleosome-free region in the vicinity of the TSS play key roles in regulating downstream gene expression. Similarly, analysis of the SOX2 promoter revealed that the nucleosome-free region near the TSS in the pSUB vector regulated by UCOE alone was considerably wider than that in the pSSB vector regulated by saRNA and in the pSUSB vector co-regulated by saRNA and UCOE. Notably, however, we detected broad similarities among these three vectors with respect to DNA methylation characteristics (Figure 5(d)).

## Discussion

### The impact of DNA methylation and nucleosome positioning within the PhiC31 integration region on gene expression

By inserting expression vectors containing OCT4 and SOX2 promoters into the PhiC31 integration site, we observed changes in promoter chromatin structure both before and after integration. These changes included alterations in the levels of DNA methylation, variations in the widths of nucleosome-free regions, shifts in nucleosome positioning, and translocation of the nucleosome-free regions.

The insertion of the SOX2 promoter within the PhiC31 integration site, specifically 300 bp upstream of the transcription start site (TSS), resulted in a 2.6-fold increase in DNA methylation levels. Moreover, the expression level of the integrated pSB vector was six times higher than that of the non-site-specifically integrated pS vector.When UCOE and saRNA were used to modulate the chromatin structure of the SOX2 promoter region in the PhiC31-integrated pSB vector, the methylation levels upstream of the TSS were reduced to the same level as that of the non-integrated pS vector with consistent SOX2 promoter methylation. Consequently, the expression level significantly decreased, suggesting that high DNA methylation within the nucleosome-free region located 300 bp upstream of the TSS in the PhiC31 integration site plays a crucial role in regulating gene expression.

As for the OCT4 gene promoter within the PhiC31 integration site, the highly expressed pOUB vector showed lower DNA methylation within the nucleosome-free region upstream of the OCT4 promoter TSS compared to the pOOB and pOUOB vectors. In this case, the high DNA methylation in pOOB and pOUOB seemed to have an inhibitory effect on expression, while the low methylation in pOUB appeared to have a positive effect. However, it should be noted that the nucleosome-free region near the TSS of the pOUB vector was noticeably wider than the other two vectors. Furthermore, when comparing the site-specifically integrated pOOB and pOUOB vectors with the non-specifically integrated pOB vector, an increase in methylation levels upstream of the TSS was observed. This increase correlated with higher expression levels determined by Western blotting. However, average fluorescence intensity analysis showed no significant changes. Nonetheless, whether the expression level increased or remained unchanged, both situations suggest that DNA methylation within the OCT4 promoter of PhiC31 site-specifically integrated vectors is not the sole determinant of downstream gene expression efficiency. Therefore, considering all the findings, it can be concluded that the expression of the OCT4 promoter within the PhiC31 integration site is likely influenced more by chromatin structure than by DNA methylation.

The DNA low methylation level is often negatively correlated with gene expression, especially in the DNA hypermethylation within a 2 kb range of the promoter region (TSS), which is closely associated with gene repression [31–34]. The relationship between genome-wide promoter methylation and gene expression depends on the CpG density of the promoter. High-density CpG promoters (greater than 65%) exhibit low methylation regardless of gene expression. Promoters with moderate CpG density (50–55%) show lower levels of methylation in highly expressed genes, while they have higher levels of methylation in lowly expressed genes. Promoters with low CpG density (less than 50%) show high methylation levels regardless of gene expression [33]. The OCT4 promoter belongs to the moderate CpG density promoter category, while SOX2 belongs to the high-density CpG promoter category. In the PhiC31 integration region, the OCT4 promoter with moderate CpG density shows higher expression when methylated at low levels (pOUB), which seems to be consistent with the pattern observed at the genome-wide level. However, the impact of DNA methylation on expression may be relatively smaller compared to chromatin structure. On the other hand, the SOX2 promoter with high CpG density shows a positive correlation between high expression and a certain degree of high methylation in the PhiC31 integration region, while low methylation levels are correlated with low expression, completely opposite to the pattern observed in the genome-wide region. The expression level of promoters with low methylation levels is mainly influenced by nucleosome positioning. Therefore, it can be seen that the influence of DNA methylation on promoter expression patterns in the PhiC31 integration region varies depending on the type of promoter.

Open chromatin generally refers to the structure of the decondensed state of chromatin in cells, which facilitates efficient and stable regulation of gene expression [31]. The expression of genes is closely associated with nucleosome-depleted regions within the promoter region, and the positioning of nucleosomes is closely reflected by occupancy [32]. The stability, structure, and binding status of core nucleosomes play pivotal roles in transcriptional regulation [33–35]. If the open chromatin region within the PhiC31 integration site is defined as a natural open chromatin region, integration within this region can lead to changes in nucleosome positioning and quantity in the promoter region, which will accordingly have an influence on gene expression.

Compared with the pSB and pSUB vectors, we detected little expression of genes encoded in the pSSB vector in the PhiC31-directed integration site, which is correlated with the dense nucleosome distribution in the downstream region adjacent to the TSS of the pSSB vector promoter. In the vector harbouring the OCT4 gene promoter, larger open chromatin regions from −600 to −1 upstream of the TSS were observed to correspond with higher expression levels. Similarly, when a UCOE was used to regulate the promoter region of the pOB vector, thereby forming a broader open chromatin region from −600 to −1, we detected a relative increase in expression from this vector. Across all vector promoter regions, regardless of whether it is from −200 to + 1 upstream of the TSS or from 0 to + 200 downstream, larger open chromatin regions are associated with higher levels of gene expression [36]. These findings thus tend to indicate that the effects of nucleosome occupancy on expression patterns within the PhiC31 integration site are characterized by consistent patterns and regulations similar to those of other genomic regions.

### Interactions between the regulatory factors saRNA and UCOE within the PhiC31 integration site

Small activating RNA (saRNA) has been shown to activate gene expression at the transcriptional level [25]. UCOE, on the other hand, is a gene element that exhibits dominant chromatin remodelling or opening functions. It prevents transgene silencing and significantly increases the median expression levels in stably transfected cells [27–28].When UCOE is used alone for regulation, the OCT4 and SOX2 promoter regions within the PhiC31 integration site form wider open chromatin regions, with a significant decrease in DNA methylation levels. When saRNA is used alone to target regulate the promoter of the vector, there is also a noticeable reduction in DNA methylation levels on the SOX2 promoter, along with clear changes in the number and positioning of nucleosomes. The DNA methylation changes are relatively minor on the OCT4 promoter, but there are significant variations in the number and positioning of nucleosomes.

When UCOE and saRNA are used together to co-regulate the PhiC31-integrated vector (pOUOB), the DNA methylation status on the OCT4 promoter is almost identical to that regulated by saRNA alone on the pOOB promoter region. Additionally, the positioning of nucleosomes and the nucleosome-free region return to the state with no regulation (pOB). In the vector mediated by the SOX2 gene promoter, the positioning and quantity of nucleosomes in the pSUSB vector, co-regulated by UCOE and saRNA, also revert to the unregulated state (pSB). Furthermore, the degree of DNA methylation is closer to the state regulated by saRNA alone.

These findings suggest that targeted saRNA on both promoters can overcome the inhibitory effect of UCOE on DNA methylation. The interplay between UCOE and saRNA leads to an antagonistic or offsetting effect on the changes in nucleosome positioning and quantity at both promoters.

### The stable and optimizable expression of vectors following PhiC31 site-specific integration

Comparatively, the pSB vector after PhiC31 site-specific integration exhibits a significant increase in expression levels, unlike the non-site-specific integrated pS vector with the SOX2 promoter. Interestingly, regardless of whether UCOE or saRNA is used for regulation, the expression level of the SOX2 promoter within the PhiC31 integration site only decreases rather than increases. From a chromatin perspective, it can be observed that a certain degree of high methylation in the upstream nucleosome-free region (NFR) and the nucleosome-free region near the transcription start site (TSS) within the integration site is most favourable for high expression.

On the other hand, the expression levels of the pOB vector, carrying the OCT4 promoter, show no significant changes after PhiC31 integration compared to the non-site-specific integrated pOCT4 vector. However, when UCOE is used to regulate the expression of pOUB within the integration site, it exhibits higher expression than pOB. Similarly, when saRNA is utilized for regulation, pOOB demonstrates higher expression than pOB. This indicates that the open chromatin structure within the PhiC31 integration site can still achieve higher expression levels when optimized through regulation by UCOE and saRNA. The optimal structures for high expression on the OCT4 promoter are the wider nucleosome-free region near the TSS in the pOUB vector and low methylation. In other words, when the chromatin structure of the promoter within the PhiC31 integration site is in its ‘optimal state’ or ‘regulatable state,’ being in the ‘optimal state’ means further regulation won’t enhance downstream gene expression, while being in the ‘regulatable state’ allows both UCOE and saRNA to increase expression.

After PhiC31 site-specific integration, the regulated vectors pOUB and pOUOB, along with the non-integrated vectors pOU and pOUO, were introduced into CHO cells. The results revealed no significant differences in expression among the four clones of the PhiC31 integrated vectors (pOUB and pOUOB). However, there was considerable variation in expression among the four clones of the non-integrated vectors (pOU and pOUO), indicating an unstable expression pattern. These findings suggest that, following PhiC31 site-specific integration, the vectors in their ‘regulatable state’ exhibit more stable expression when regulated by UCOE or saRNA.

## Conclusions

In this study, we established that insertion of expression vectors containing the SOX2 and OCT4 promoters in the PhiC31 integration site results in either upregulated expression or complete inactivation. Within the PhiC31 integration site, the SOX2 promoter forms a chromatin structure that is most favourable for high expression, characterized by a certain degree of DNA methylation and a nucleosome-free region near the transcription start site. Contrastingly, DNA methylation of the OCT4 promoter within the PhiC31 integration site has a weaker association with expression. The efficiency of expression under the control of the OCT4 promoter is associated with the width of the nucleosome-free region near the transcription start site. In response to regulation by saRNA and UCOE, levels of expression mediated by the OCT4 promoter within the PhiC31 integration site can be upregulated. In contrast, expression under the control of the SOX2 promoter is suppressed to varying degrees by saRNA and UCOE.

## Supplementary Material

Supplementary table 1 Primers for nucleosome deletion analys.docx

## Data Availability

Data will be available from the authors upon request.
