# Supplementary material for: Study on chromatin regulation patterns of expression vectors in the PhiC31 integration site
Source: Epigenetics. 2024 Apr 9;19(1):2337085. doi: 10.1080/15592294.2024.2337085 (PMC11008548; doi:10.1080/15592294.2024.2337085)
Supplement: Supplementary table 1 Primers for nucleosome deletion analys.docx [file KEPI_A_2337085_SM2836.docx]

Supplementary table 1 Primer sequences for analysis of nucleosomes deletion

| Name | sequence（5' to 3'） | length（bp） |
| --- | --- | --- |
| 1-OCT4 H3-F | TGATGCATTGAGGGATAGCG | 20 |
| 1-OCT4 H3-R | GTTCTTCAGGAACCCAGGTG | 20 |
| 2-OCT4 H3-F | CCAAAGCGGGTGTCTTATCA | 20 |
| 2-OCT4 H3-R | TGATAAGACACCCGCTTTGG | 20 |
| 3-OCT4 H3-F | CCAAAGCGGGTGTCTTATCA | 20 |
| 3-OCT4 H3-R | CGCTATCCCTCAATGCATCA | 20 |
| 4-OCT4 H3-F | AACATCCTTCGCCTCAGTTT | 20 |
| 4-OCT4 H3-R | TTGCGAAGGGACTACTCAAC | 20 |
| 5-OCT4 H3-F | TATAGCACGGAGGCCTTGTC | 20 |
| 5-OCT4 H3-R | CGATACTGGCCAAATCCAGC | 20 |
| 6-OCT4 H3-F | GTATCGGGATGGGAATGCCT | 20 |
| 6-OCT4 H3-R | CACCCTCTCAGCTCCTCAAA | 20 |
| 7-OCT4 H3-F | TGAGGCCCAGTCAGTCCAAA | 20 |
| 7-OCT4 H3-R | AGACAAGGCCTCCGTGCTAT | 20 |
| 8-OCT4 H3-F | CTGAAGAACATGGAGGTGTG | 20 |
| 8-OCT4 H3-R | CTTTCAACTCCCAACCCGC | 19 |
| 9-OCT4 H3-F | CTGCACTGAGGTCCTGGAG | 19 |
| 9-OCT4 H3-R | GGAGAAACTGAGGCGAAGGA | 20 |
| 10-OCT4 H3-F | GGGAGTGATTCCAGACAG | 18 |
| 10-OCT4 H3-R | CATGATTAAAGGCGTGAG | 18 |
| 11-OCT4 H3-F | CGGGTTGGGAGTTGAAAG | 18 |
| 11-OCT4 H3-R | GGACCACTGTGCCCTGTT | 18 |
| 12-OCT4 H3-F | GTGGTCCAAGCCTGTAGT | 18 |
| 12-OCT4 H3-R | CAGGGTCTCACTTTGTTGC | 19 |
| 13-OCT4 H3-F | AGCCATCATTGTACTCCAC | 19 |
| 13-OCT4 H3-R | CAATGGTGTCTGTGGAAGG | 19 |
| 1-SOX2 H3-F | CGAGAGGGGATACAAAGGTT | 20 |
| 1-SOX2 H3-R | TGCCGGGTTTTGCATGA | 17 |
| 2-SOX2 H3-F | TTCATGCAAAACCCGGCA | 18 |
| 2-SOX2 H3-R | GGGGCTGTCAGGGAATAAAT | 20 |
| 3-SOX2 H3-F | TAATAACAATCATCGGCGGC | 20 |
| 3-SOX2 H3-R | GGCGAAGAATAATTTGGGGG | 20 |
| 4-SOX2 H3-F | GTGTTTGCAAAAGGGGGAAA | 20 |
| 4-SOX2 H3-R | GCCGCCGATGATTGTTATTATT | 22 |
| 5-SOX2 H3-F | CCCAAATTATTCTTCGCCTGA | 21 |
| 5-SOX2 H3-R | CTCCATCATGTTGTACATGCG | 21 |
| 6-SOX2 H3-F | ACAACATGATGGAGACGGAG | 20 |
| 6-SOX2 H3-R | GGTCCGGGCTGTTTTTCT | 18 |
| 7-SOX2 H3-F | GGTTTGGGTCTCCTAACTTCT | 21 |
| 7-SOX2 H3-R | CATTGTTCTCCCGCTCATC | 19 |
| 8-SOX2 H3-F | GAGCGGGAGAACAATGACA | 19 |
| 8-SOX2 H3-R | GCAGGGTACTTAAATGAGGATG | 22 |
| 9-SOX2 H3-F | GCGTCCCATCCTCATTTA | 18 |
| 9-SOX2 H3-R | TCTCGCAGCAACAGGTCA | 18 |
| 10-SOX2 H3-F | CCCGTCACATGGATGGTT | 18 |
| 10-SOX2 H3-R | GTCTTAAAGAGGCAGCAAACTA | 22 |

Supplementary table 2 QC results of cleaned reads filtered from raw data

| Sample_ID | RawReadsNum | RawBaseNum | RawQ20Rate | RawQ30Rate | AdapterCleanBaseRate | CleanReadsNum | CleanBaseNum | CleanQ20Rate | CleanQ30Rate | CleanReadsRate | CleanBaseRate | CleanData(Gb) |
| --- | --- | --- | --- | --- | --- | --- | --- | --- | --- | --- | --- | --- |
| S | 1065826 | 159873900 | 90.41% | 80.07% | 92.57% | 1064094 | 147886474 | 90.02% | 79.37% | 99.84% | 92.50% | 0.148 |
| SB | 733610 | 110041500 | 91.45% | 82.49% | 92.00% | 732128 | 101137772 | 91.28% | 82.15% | 99.80% | 91.91% | 0.101 |
| SS | 643856 | 96578400 | 93.87% | 86.03% | 85.88% | 641850 | 82800341 | 93.45% | 85.37% | 99.69% | 85.73% | 0.083 |
| SSB | 779700 | 116955000 | 89.84% | 80.34% | 93.66% | 779014 | 109492206 | 89.76% | 80.13% | 99.91% | 93.62% | 0.109 |
| SUB | 847060 | 127059000 | 90.89% | 81.83% | 90.75% | 844834 | 115151029 | 90.55% | 81.26% | 99.74% | 90.63% | 0.115 |
| SUSB | 675040 | 101256000 | 85.33% | 74.36% | 91.34% | 668060 | 92189563 | 85.28% | 74.08% | 98.97% | 91.05% | 0.092 |

Supplementary table 3 QC results of cleaned reads filtered from raw data

| \| ample  ID \| RawReadsNum \| RawBaseNum \| RawQ20Rate \| RawQ30Rate \| Adapter  Clean  BaseRate \| CleanReadsNum \| CleanBaseNum \| Clean  Q20Rate \| Clean  Q30Rate \| CleanReadsRate \| CleanBaseRate \| CleanData(Gb) \| \| --- \| --- \| --- \| --- \| --- \| --- \| --- \| --- \| --- \| --- \| --- \| --- \| --- \| \| O \| 881644 \| 132246600 \| 93.13% \| 85.40% \| 94.14% \| 880352 \| 124461942 \| 93.05% \| 85.22% \| 99.85% \| 94.11% \| 0.124 \| \| OB \| 1042206 \| 156330900 \| 92.42% \| 84.26% \| 94.16% \| 1041296 \| 147171916 \| 92.28% \| 83.95% \| 99.91% \| 94.14% \| 0.147 \| \| OO \| 691298 \| 103694700 \| 91.26% \| 82.88% \| 93.21% \| 688908 \| 96602437 \| 91.33% \| 82.83% \| 99.65% \| 93.16% \| 0.097 \| \| OOB \| 272800 \| 40920000 \| 92.39% \| 84.39% \| 93.73% \| 271480 \| 38337490 \| 92.29% \| 84.15% \| 99.52% \| 93.69% \| 0.038 \| \| OUOB \| 1075538 \| 161330700 \| 92.95% \| 85.77% \| 94.16% \| 1074280 \| 151877675 \| 92.79% \| 85.49% \| 99.88% \| 94.14% \| 0.152 \| \| OUB \| 752694 \| 112904100 \| 92.18% \| 84.42% \| 93.86% \| 749826 \| 105881959 \| 92.12% \| 84.21% \| 99.62% \| 93.78% \| 0.106 \| |  |  |  |  |  |  |  |  |  |  |  |  |
| --- | --- | --- | --- | --- | --- | --- | --- | --- | --- | --- | --- | --- | --- | --- | --- | --- | --- | --- | --- | --- | --- | --- | --- | --- | --- | --- | --- | --- | --- | --- | --- | --- | --- | --- | --- | --- | --- | --- | --- | --- | --- | --- | --- | --- | --- | --- | --- | --- | --- | --- | --- | --- | --- | --- | --- | --- | --- | --- | --- | --- | --- | --- | --- | --- | --- | --- | --- | --- | --- | --- | --- | --- | --- | --- | --- | --- | --- | --- | --- | --- | --- | --- | --- | --- | --- | --- | --- | --- | --- | --- | --- | --- | --- | --- | --- | --- | --- | --- | --- | --- | --- | --- | --- |
|  |  |  |  |  |  |  |  |  |  |  |  |  |
|  |  |  |  |  |  |  |  |  |  |  |  |  |
|  |  |  |  |  |  |  |  |  |  |  |  |  |
|  |  |  |  |  |  |  |  |  |  |  |  |  |
|  |  |  |  |  |  |  |  |  |  |  |  |  |
| SaRNA sequence  O-38  S278  OCT4 promoter sequence  AGAACGGG**GC**CTACCGTGGTATTAGATGTCTGAGTTTTGGTTGAGAGGGG  A**GC**AAGGAACCTGATGT**GC**AGGTTCCATAGTGGAGGGG**GC**CCAAA**GC**GGG  TGTCTTATCACTCTGTTTCA**GC**AAAGGTTGGGAAACTGAG**GC**CCAGTCAG  TCCAAAGTCTGGTCCCTTGAAGGGGAAGTAGGGACCAACCCCTTAGTCTG  TTAGATGAGGAGAGTCTGGAGTCTGATTCTGGAAGACGGAGGGGTGGGGG  GATGGGGGGGTGGGGGGATATA**GC**ACGGAG**GC**CTTGTCTG**GC**AGTCTACT  CTTGAAGATGGGGTGAAATTTG**GC**AG**GC**TGG**GC**AGATGGT**GC**CAG**GC**ACC  CAG**GC**TGCGGGGTG**GC**TGGATTTG**GC**CAGTATCGGGATGGGAAT**GC**CTAG  GATTCTGGATGGAT**CG**GGGGAAG**GC**ATAAGGGA**GC**A**GC**TG**GC**CATTGT**G**C  TTATG**GC**TGTTGAT**GC**ATTGAGGGATA**GCGC**CACACACACATTCAATAAA  TTTGAGGA**GC**TGAGAGGGTGACTG**GC**CCCTGAAG**GC**ACAGT**GC**CAGAGGT  CTGTGGAGAGGGGGTCAA**GC**ACCTGGGTTCCTGAAGAACATGGAGGTGTG  GGAGTGATTCCAGACA**GC**TGGGATGT**GC**AGA**GC**CTGAGAGAGT**GC**CAGGG  A**GC**GGGTTGGGAGTTGAAAGTTGGGTGTGGTG**GC**TCAC**GC**CTTTAATCAT  GACACTGG**GC**G**GC**AGAG**GC**GGGAGGATTTCTTGAGGACAGGAATTCAAGA  CCA**GC**CTGGGTAACATA**GC**AAG**GC**CCCATCTCTACTAAAAATAAAAAAAC  TAACAGG**GC**ACAGTGGTCCAA**GC**CTGTAGTCCCA**GC**CACTTAGGAG**GC**TG  GA**GC**AGAAGGATT**GC**TTTG**GC**CCAGTAGATCGAG**GC**TACATTGA**GC**CATC  ATTGTACTCCACT**GC**ACTCCAGTCTGG**GC**AACAAAGTGAGACCCTGTCTT  AAAAAATAAAAATAAAAAAAGTTTCTGTGGGGGACCT**GC**ACTGAGGTCCT  GGAGGGGC**GC**CAGTTGTGTCTCCCGGTTTTCCCCTTCCACAGACACCATT  **GC**CACCACCATTAG**GC**AAACATCCTTC**GC**CTCAGTTTCTCCCCCCACCTC  CCTCTCCTCCACCCATCCAGGGG**GC**GGG**GC**CAGAGGTCAAG**GC**TAGTGGG  TGGGACTGGGGAGGGAGAGAGGGGTTGAGTAGTCCCTTCGCAAGCCCTCA  TTTCACCAG**GC**CCCCG**GC**TTGGGGC**GC**CTTCCTTCCCCATGGCGGGACAC  CTG**GC**TTCGGATTTC**GC**CTTCTCG**C**CCCC  SOX2 promoter sequence  ACTTAGGCCCTCCGCGCCTACAGCTCAAGCCACATCCGAAGGGGGAGGGAGCCGGGAGCTGCGCGCGGGGCCGCCGGGGGGAGGGGTGGCACCGCCCACGCCGGGCGGCCACGAAGGGCGGGGCAGCGGGCGCGCGCGCGGCGGGGGGAGGGGCCGGCGCCGCGCCCGCTGGGAATTGGGGCCCTAGGGGGAGGGCGGAGGCGCCGACGACCGCGGCACTTACCGTTCGCGGCGTGGCGCCCGGTGGTCCCCAAGGGGAGGGAAGGGGGAGGCGGGGCGAGGACAGTGACCGGAGTCTCCTCAGCGGTGGCTTTTCTGCTTGGCAGCCTCAGCGGCTGGCGCCAAAACCGGACTCCGCCCACTTCCTCGCCCGCCGGTGCGAGGGTGTGGAATCCTCCAGACGCTGGGGGAGGGGGAGTTGGGAGCTTAAAAACTAGTACCCCTTTGGGACCACTTTCAGCAGCGAACTCTCCTGTACACCAGGGGTCAGTTCCACAGACGCGGGCCAGGGGTGGGTCATTGCGGCGTGAACAATAATTTGACTAGAAGTTGATTCGGGTGTTTCCGGAAGGGGCCGAGTCAATCCGCCGAGTTGGGGCACGGAAAACAAAAAGGGAAGGCTACTAAGATTTTTCTGGCGGGGGTTATCATTGGCGTAACTGCAGGGACCACCTCCCGGGTTGAGGGGGCTGGATCTCCAGGCTGCGGATTAAGCCCCTCCCGTCGGCGTTAATTTCAAACTGCGCGACGTTTCTCACCTGCCTTCGCCAAGGCAGGGGCCGGGACCCTATTCCAAGAGGTAGTAACTAGCAGGACTCTAGCCTTCCGCAATTCATTGAGCGCATTTACGGAAGTAACGTCGGGTACTGTCTCTGGCCGCAAGGGTGGGAGGAGTACGCATTTGGCGTAAGGTGGGGCGTAGAGCCTTCCCGCCATTGGCGGCGGATAGGGCGTTTACGCGACGGCCTGACGTAGCGGAAGACGCCTTAGTGGGGGGGAAGGTTCTAGAAAAGCGGCGGCAGCGGCTCTAGCGGCAGTAGCAGCAGCGCCGGGTCCCGTGCGGAGGTGCTCCTCGCAGAGTTGTTTCTCCAGCAGCGGCAGTTCTCACTACAGCGCCAGGACGAGTCCGGTTCGTGTTCGTCCGCGGAGATCTCTCTCATCTCGCTCGGCTGCGGGAAATCGGGCTGAAGCGACTGAGTCCGCGATGGAGGTAACGGGTTTGAAATCAATGAGTTATTGAAAAGGGCATGGCGAGGCCGTTGGCGCCTCAGTGGAAGTCGGCCAGCCGCCTCCGTGGGAGAGAGGCAGGAAATCGGACCAATTCAGTAGCAGTGGGGCTTAAGGTTTATGAACGGGGTCTTGAGCGGAGGCCTGAGCGTACAAACAGCTTCCCCACCCTCAGCCTCCCGGCGCCATTTCCCTTCACTGGGGGTGGGGGATGGGGAGCTTTCACATGGCGGACGCTGCCCCGCTGGGGTGAAAGTGGGGCGCGGAGGCGGGACTTCTTATTCCCTTTCTAAAGCACGCTGCTTCGGGGGCCACGGCGTCTCCTCGGTAAGTCTCGAG  1.5kb UCOE Sequence  ACTTAGGCCCTCCGCGCCTACAGCTCAAGCCACATCCGAAGGGGGAGGGAGCCGGGAGCTGCGCGCGGGGCCGCCGGGGGGAGGGGTGGCACCGCCCACGCCGGGCGGCCACGAAGGGCGGGGCAGCGGGCGCGCGCGCGGCGGGGGGAGGGGCCGGCGCCGCGCCCGCTGGGAATTGGGGCCCTAGGGGGAGGGCGGAGGCGCCGACGACCGCGGCACTTACCGTTCGCGGCGTGGCGCCCGGTGGTCCCCAAGGGGAGGGAAGGGGGAGGCGGGGCGAGGACAGTGACCGGAGTCTCCTCAGCGGTGGCTTTTCTGCTTGGCAGCCTCAGCGGCTGGCGCCAAAACCGGACTCCGCCCACTTCCTCGCCCGCCGGTGCGAGGGTGTGGAATCCTCCAGACGCTGGGGGAGGGGGAGTTGGGAGCTTAAAAACTAGTACCCCTTTGGGACCACTTTCAGCAGCGAACTCTCCTGTACACCAGGGGTCAGTTCCACAGACGCGGGCCAGGGGTGGGTCATTGCGGCGTGAACAATAATTTGACTAGAAGTTGATTCGGGTGTTTCCGGAAGGGGCCGAGTCAATCCGCCGAGTTGGGGCACGGAAAACAAAAAGGGAAGGCTACTAAGATTTTTCTGGCGGGGGTTATCATTGGCGTAACTGCAGGGACCACCTCCCGGGTTGAGGGGGCTGGATCTCCAGGCTGCGGATTAAGCCCCTCCCGTCGGCGTTAATTTCAAACTGCGCGACGTTTCTCACCTGCCTTCGCCAAGGCAGGGGCCGGGACCCTATTCCAAGAGGTAGTAACTAGCAGGACTCTAGCCTTCCGCAATTCATTGAGCGCATTTACGGAAGTAACGTCGGGTACTGTCTCTGGCCGCAAGGGTGGGAGGAGTACGCATTTGGCGTAAGGTGGGGCGTAGAGCCTTCCCGCCATTGGCGGCGGATAGGGCGTTTACGCGACGGCCTGACGTAGCGGAAGACGCCTTAGTGGGGGGGAAGGTTCTAGAAAAGCGGCGGCAGCGGCTCTAGCGGCAGTAGCAGCAGCGCCGGGTCCCGTGCGGAGGTGCTCCTCGCAGAGTTGTTTCTCCAGCAGCGGCAGTTCTCACTACAGCGCCAGGACGAGTCCGGTTCGTGTTCGTCCGCGGAGATCTCTCTCATCTCGCTCGGCTGCGGGAAATCGGGCTGAAGCGACTGAGTCCGCGATGGAGGTAACGGGTTTGAAATCAATGAGTTATTGAAAAGGGCATGGCGAGGCCGTTGGCGCCTCAGTGGAAGTCGGCCAGCCGCCTCCGTGGGAGAGAGGCAGGAAATCGGACCAATTCAGTAGCAGTGGGGCTTAAGGTTTATGAACGGGGTCTTGAGCGGAGGCCTGAGCGTACAAACAGCTTCCCCACCCTCAGCCTCCCGGCGCCATTTCCCTTCACTGGGGGTGGGGGATGGGGAGCTTTCACATGGCGGACGCTGCCCCGCTGGGGTGAAAGTGGGGCGCGGAGGCGGGACTTCTTATTCCCTTTCTAAAGCACGCTGCTTCGGGGGCCACGGCGTCTCCTCGGTAAGTCTCGAG |  |  |  |  |  |  |  |  |  |  |  |  |
